# Integrated analysis uncovers *KCMF1* genetic susceptibility and the *SNRPD2* axis in renal cell carcinoma

**DOI:** 10.7150/ijms.129880

**Published:** 2026-02-18

**Authors:** Jiunn-Bey Pao, Yu-Mei Hsueh, Pei-Ling Chen, Tzu-Hao Huang, Chia-Yang Li, Jiun-Hung Geng, Te-Ling Lu, Chao-Yuan Huang, Shu-Pin Huang, Bo-Ying Bao

**Affiliations:** 1 Department of Pharmacy, Taipei City Hospital, Taipei 108, Taiwan.; 2 School of Pharmacy, National Defense Medical University, Taipei 114, Taiwan.; 3 Department of Family Medicine, Wan Fang Hospital, Taipei Medical University, Taipei 110, Taiwan.; 4 Department of Public Health, School of Medicine, College of Medicine, Taipei Medical University, Taipei 110, Taiwan.; 5 Department of Urology, National Taiwan University Hospital, College of Medicine, National Taiwan University, Taipei 100, Taiwan.; 6 Department of Urology, Taipei Veterans General Hospital, Taipei 112, Taiwan.; 7 Department of Urology, College of Medicine, National Yang Ming Chiao Tung University, Taipei 112, Taiwan.; 8 Shu-Tien Urological Science Research Center, National Yang Ming Chiao Tung University, Taipei 112, Taiwan.; 9 Graduate Institute of Medicine, College of Medicine, Kaohsiung Medical University, Kaohsiung 807, Taiwan.; 10 Department of Medical Research, Kaohsiung Medical University Hospital, Kaohsiung 807, Taiwan.; 11 Department of Urology, Kaohsiung Medical University Hospital, Kaohsiung 807, Taiwan.; 12 Department of Urology, Kaohsiung Municipal Hsiao-Kang Hospital, Kaohsiung 812, Taiwan.; 13 Department of Pharmacy, China Medical University, Taichung 406, Taiwan.; 14 Graduate Institute of Clinical Medicine, College of Medicine, Kaohsiung Medical University, Kaohsiung 807, Taiwan.; 15 Institute of Medical Science and Technology, College of Medicine, National Sun Yat-Sen University, Kaohsiung 804, Taiwan.

**Keywords:** genetic variants, renal cell carcinoma, prognosis, weighted gene co-expression network analysis, ZZ-type zinc finger family

## Abstract

The ZZ-type zinc-finger family has emerged as an important regulator of tumorigenesis; however, its roles in renal cell carcinoma (RCC) susceptibility and prognosis are largely unexplored. This study aimed to systematically evaluate the genetic variants within this gene family to identify novel risk drivers and elucidate their downstream pathogenic networks. A total of 148 single-nucleotide polymorphisms (SNPs) were genotyped across 17 ZZ-type zinc finger genes in a cohort of 630 Taiwanese participants (312 patients with RCC and 318 healthy controls). The results were validated using pooled transcriptomic analysis of 18 independent datasets. Weighted gene co-expression network analysis (WGCNA) was used to construct tumor-specific networks and identify key driver genes. The Asian-specific variant of *KCMF1* rs146409312 emerged as a significant susceptibility locus. The minor A allele conferred a 3.38-fold increased risk of RCC (adjusted odds ratio = 3.22, 95% confidence interval = 1.57-6.58, *p* = 0.001). *KCMF1* was consistently upregulated in tumor tissues and was associated with poor patient survival. WGCNA identified a clinically relevant *KCMF1*-associated gene module enriched in ribosomal biogenesis and MYC target signaling. Within this network, *SNRPD2* was identified as a critical hub gene, and its overexpression was strongly correlated with advanced tumor grade, stage, and reduced overall survival (*p* < 0.001). In conclusion, *KCMF1* rs146409312 was identified as a potent, population-specific risk factor for RCC. A pathogenic *KCMF1*-driven network converging on *SNRPD2* was delineated, offering novel insights into RCC etiology and highlighting potential biomarkers for prognostic stratification.

## Introduction

Renal cell carcinoma (RCC) represents an important and escalating global health challenge, accounting for 2%-3% of all adult malignancies, and has a rising incidence in developed nations 1. Despite the elucidation of the von Hippel-Lindau (VHL) tumor suppressor pathway as a cardinal driver in clear-cell RCC, the clinical trajectory of patients remains highly variable 2. Contemporary management of metastatic RCC has evolved rapidly from cytokine-based regimens to targeted therapies that inhibit vascular endothelial growth factor and the mammalian target of rapamycin and, more recently, to immune checkpoint inhibitors 3. Although these modalities have extended overall survival, primary and acquired resistance remains a pervasive issue, with 5-year survival rates for distant metastatic disease of ~12% 4. Current prognostic models primarily utilize clinicopathological factors, such as tumor stage and grade, with well-established driver mutations in VHL tumor suppressor and chromatin remodeling genes, such as polybromo 1, BRCA1 associated deubiquitinase 1, and SET domain containing 2 histone lysine methyltransferases 5. However, these markers often fail to capture the full biological heterogeneity of the disease, and the identification of robust biomarkers to predict the therapeutic response and long-term survival remains a critical unmet need in the management of RCC 6.

The ZZ-type zinc finger domain is a unique structural motif consisting of 40-60 amino acids that coordinates two zinc ions and functions primarily as a mediator of protein-protein interactions and chromatin recognition 7. Genes harboring this domain, such as the transcriptional co-activators, CREB binding lysine acetyltransferase (CREBBP) and EP300 lysine acetyltransferase (EP300); the autophagy receptors, sequestosome 1 (SQSTM1) and NBR1 autophagy cargo receptor (NBR1); and the E3 ubiquitin ligases, HECT and RLD domain containing E3 ubiquitin protein ligase 2 (HERC2), MIB E3 ubiquitin protein ligase 1 (MIB1), and MIB2; are increasingly recognized as pivotal regulators of cellular homeostasis, transcriptional integrity, and proteostasis 7. Dysregulation of these ZZ-domain proteins contributes to carcinogenesis through diverse mechanisms, including aberrant histone acetylation, disruption of autophagic flux, and modulation of Notch and nuclear factor-κB signaling pathways 8.

In the context of RCC, the amplification of chromosome 5q, a frequent event in clear-cell RCC, leads to the overexpression of SQSTM1, which drives oxidative stress resistance 9. Conversely, the downregulation of NBR1 has been correlated with poor prognosis and sunitinib resistance 10, whereas the potassium channel modulatory factor 1 (KCMF1)-fused in sarcoma (FUS) axis has recently been implicated in metastasis via JUN N-terminal kinase (JNK) signaling 11. Furthermore, genetic variants of chromatin modifiers, such as EP300 and CREBBP, have been implicated in broad cancer susceptibility 12, 13, suggesting that genetic variants within this superfamily may influence tumor etiology and progression.

Despite the emerging significance of ZZ-type zinc-finger family genes in oncology, their roles in RCC are unclear. Therefore, this study systematically evaluated the association between genetic variants in 17 ZZ-type zinc-finger family genes and the risk of RCC in a cohort of 630 Taiwanese participants. Transcriptomic and weighted gene co-expression network analyses (WGCNA) were integrated to validate the clinical relevance of the identified candidate, *KCMF1*. By bridging germline genetics with tumor expression profiles, critical downstream hub genes were identified and the potential pathogenic mechanisms underlying RCC progression were elucidated.

## Patients and Methods

### Study population

The study cohort comprised 630 participants recruited from three medical centers in Taipei: National Taiwan University, Taipei Medical University, and Taipei Municipal Wan Fang hospitals. The case group included 312 patients with pathologically-confirmed RCC. These patients were matched for age (± 1 y) and sex, with 318 cancer-free controls, as previously described 14-16. Demographic data were collected using structured in-person interviews, and clinical and pathological data were retrieved from medical records. All participants provided written informed consent prior to interviews and specimen collection. This study adhered to the principles of the Declaration of Helsinki and was approved by the Research Ethics Committee of National Taiwan University Hospital (approval no. 9100201527). Case and control groups were well matched for sex, age, body mass index (BMI), and smoking status (Table S1). However, differences were observed in alcohol consumption and prevalence of hypertension and diabetes (*p* < 0.001). Clinically, most patients with RCC present with early stage (Stage I-II: 81.4%) and low-grade (Grade I-II: 75.2%) tumors.

### Single-nucleotide polymorphism (SNP) selection and genotyping

Haplotype-tagging SNPs for 17 ZZ-type zinc finger family genes were selected [*CREBBP*, dystrobrevin alpha (*DTNA*), dystrobrevin beta (*DTNB*), dystrotelin (*DYTN*), *EP300*, *HERC2*, *NBR1*, *SQSTM1*, transcriptional adaptor 2A (*TADA2A*), *TADA2B*, utrophin (*UTRN*), *KCMF1*, zinc finger SWIM-type containing 2 (*ZSWIM2*), zinc finger ZZ-type containing 3 (*ZZZ3*), zinc finger ZZ-type and EF-hand domain containing 1 (*ZZEF1*), *MIB1*, and *MIB2*]. Selection was based on Han Chinese data from the 1000 Genomes Project using Haploview software, applying a linkage disequilibrium threshold of r^2^ > 0.8 and a minor allele frequency (MAF) >0.03 17, 18. Genomic DNA was extracted from peripheral blood leukocytes using a QIAamp DNA Blood Mini Kit (Qiagen) according to the manufacturer's instructions. Genotyping of the identified SNPs was performed using Affymetrix Axiom Genotyping Arrays at the National Centre for Genome Medicine, Taiwan 19. Quality control criteria excluded SNPs with call rates <98%, MAF <0.03, or significant deviations from the Hardy-Weinberg equilibrium (*p* < 0.003). A total of 148 SNPs were retained for the final analysis.

### Bioinformatic analyses

Functional annotation of the SNPs was conducted using HaploReg 20. Associations between SNPs and gene expression (expression quantitative trait loci, eQTL) were evaluated using mRNA expression data from lymphoblastoid cell lines of 158 HapMap East Asian individuals obtained from ArrayExpress (accession no. E-MTAB-264) 21. Additionally, to assess the prognostic value of gene expression, 18 publicly-available kidney cancer datasets retrieved from ArrayExpress, Gene Expression Omnibus, and The Cancer Genome Atlas (TCGA) were analyzed.

### Weighted gene co-expression network analysis (WGCNA)

To elucidate the molecular mechanisms associated with *KCMF1*, RNA-sequencing data from the TCGA-kidney renal clear cell carcinoma (KIRC) cohort were used. Patients were stratified into high- and low-*KCMF1* expression groups based on the median expression values. Differentially expressed genes (DEGs) were identified using the *limma* package (v3.64.1) in R 22. A weighted gene co-expression network was constructed for the 7,906 identified DEGs using the WGCNA package (v1.73) 23. An adjacency matrix was calculated and converted into a topological overlap matrix (TOM) to measure the gene connectivity. Hierarchical clustering based on dissimilarity (1 - TOM) was conducted to identify gene modules, with the minimum module size set to 150 genes. To determine clinical relevance, the correlation between module eigengenes and clinical traits was calculated. Gene significance (GS) was defined as the correlation between individual gene expression and clinical traits, whereas module significance was calculated as the average GS of all genes within a specific module.

### Functional annotation of module genes

To elucidate the biological functions and signaling cascades associated with the identified gene modules, functional enrichment analyses were conducted using the *clusterProfiler* package (v4.18.2) in R 24. Specifically, genes within the key modules were annotated using Gene Ontology (GO) terms, encompassing biological processes, cellular components, and molecular functions, and the Hallmark gene set collection. The top five most significantly enriched terms for both GO and Hallmark pathways were selected for visualization to characterize the coordinated biological themes of the module genes.

### Statistical analyses

The association between genetic variants and RCC risk was evaluated using logistic regression to calculate odds ratios (ORs) and 95% confidence intervals (CIs). Associations between small nuclear ribonucleoprotein D2 polypeptide (*SNRPD2*) expression and clinicopathological characteristics were assessed using Spearman's rank correlation. Survival outcomes based on *SNRPD2* expression were analyzed using Kaplan-Meier curves and log-rank tests. Statistical significance was defined at *p* < 0.05. The false discovery rate (*q*-value) was calculated to control for multiple hypothesis testing 25. These analyses were conducted using IBM SPSS Statistics for Windows, version 19.0 (IBM). For the pooled analysis, the pooled standardized mean difference (SMD) and hazard ratios (HRs) with 95% CIs were calculated using a random-effects model in Review Manager (v5.4.1, Cochrane).

## Results

**Figure 1:**
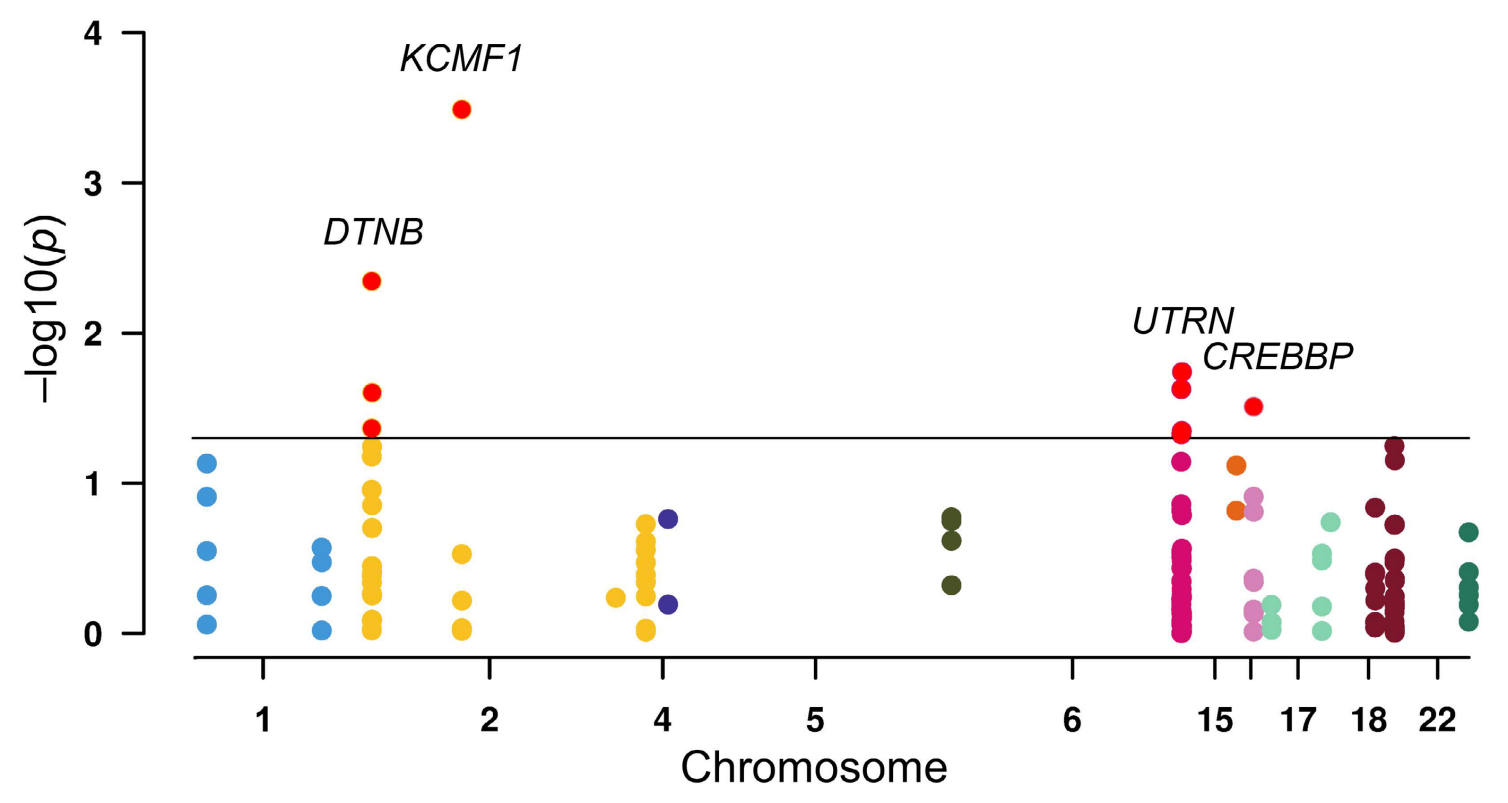
Association between ZZ-type zinc-finger family gene variants and renal cell carcinoma risk. Manhattan plot displaying the results of logistic regression analysis of 148 single-nucleotide polymorphisms (SNPs) across 17 ZZ-type zinc-finger genes. The *y*-axis represents the -log_10_(*p*) value and the *x*-axis indicates the chromosomal position of each SNP. Horizontal black line indicates a nominal significance threshold of *p* = 0.05. The SNPs that reached statistical significance are indicated in red.

To investigate the susceptibility of ZZ-type zinc-finger family genes to RCC, logistic regression analysis of 148 SNPs across the gene family was conducted (Figure 1). Initial screening indicated nominal associations (*p* < 0.05) between RCC risk and nine SNPs: three in *DTNB* (rs12986453, rs17046986, and rs11684202), one in *KCMF1* (rs146409312), four in *UTRN* (rs4243470, rs9484874, rs9376842, and rs6903007), and one in *CREBBP* (rs10454706). After multiple correction tests, the strongest signal was observed for *KCMF1* rs146409312 (*q* = 0.048). The minor A allele of rs146409312 was associated with a 3.38-fold increased risk of RCC (OR = 3.38, 95% CI = 1.74-6.58, *p* = 0.0003; Table 1). This susceptibility remained after adjusting for covariates including age, sex, BMI, smoking status, alcohol consumption, and histories of diabetes and hypertension (adjusted OR = 3.22, 95% CI = 1.57-6.58, *p* = 0.001; Table 1).

**Figure 2:**
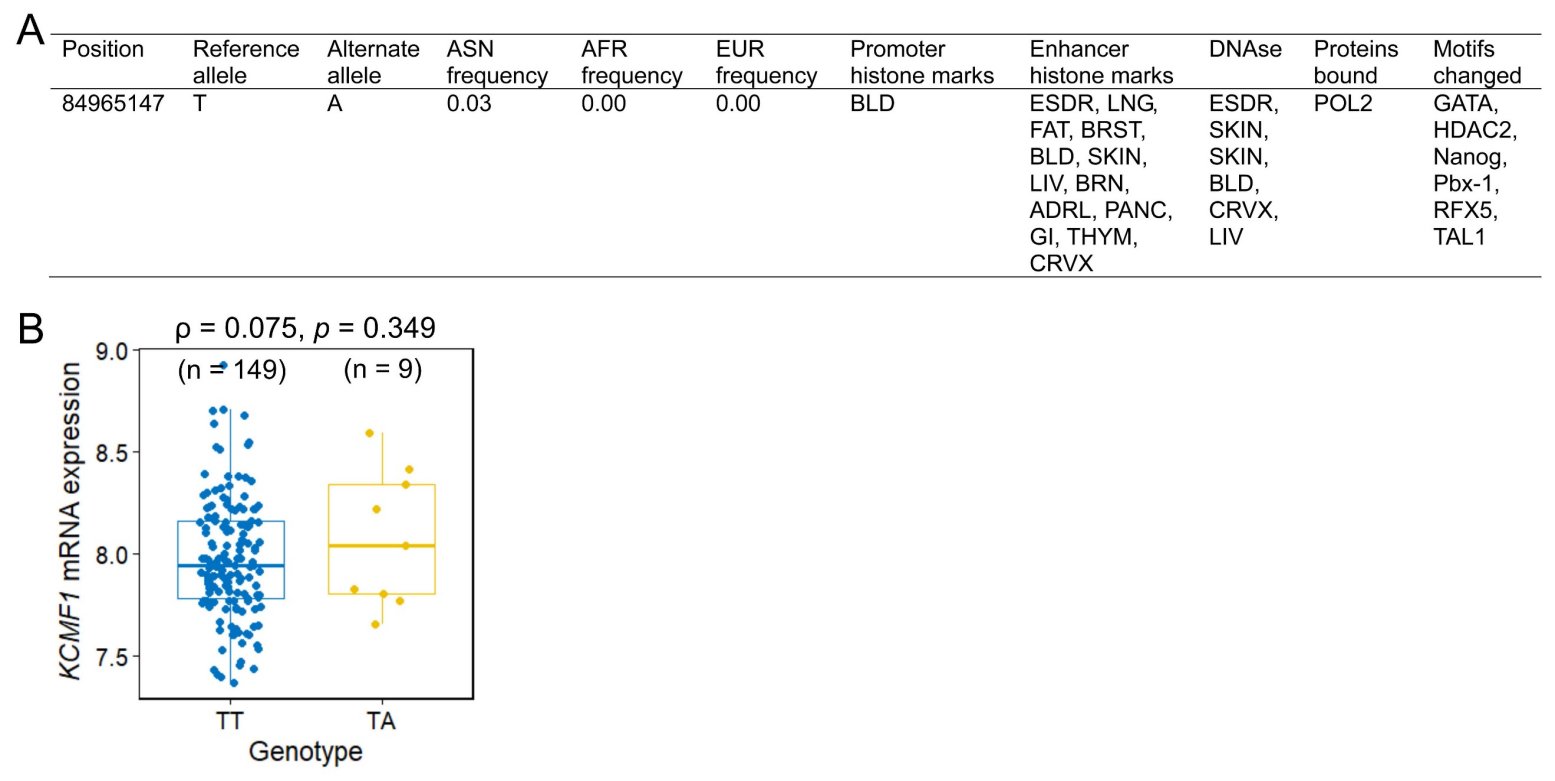
Functional annotation and expression quantitative trait loci (eQTL) analysis of *KCMF1* rs146409312. (A) HaploReg annotation illustrating the regulatory landscape of the rs146409312 locus, including the promoter/enhancer marks and transcription factor binding sites. (B) eQTL analysis correlating the rs146409312 genotype with *KCMF1* mRNA expression levels in lymphoblastoid cell lines from 158 HapMap East Asian individuals. Sample sizes for each genotype are shown in parentheses.

The functional implications of rs146409312 were assessed using *in silico* tools and eQTL analysis. HaploReg annotation indicated that rs146409312 resided within the potential promoter or enhancer regions characterized by DNase I hypersensitivity, RNA polymerase II binding, and alteration of multiple transcription factor-binding motifs (Figure 2A). Notably, rs146409312 appeared to be an Asian-specific variant; it is present in Asian population (MAF = 0.03; Figure 2A) but is absent in African, American, and European populations according to 1000 Genomes Project data. Consistent with this population specificity, in lymphoblastoid cell lines from 158 HapMap East Asian individuals, the risk allele A tended toward increased *KCMF1* mRNA expression (ρ = 0.075, *p* = 0.349; Figure 2B).

**Figure 3:**
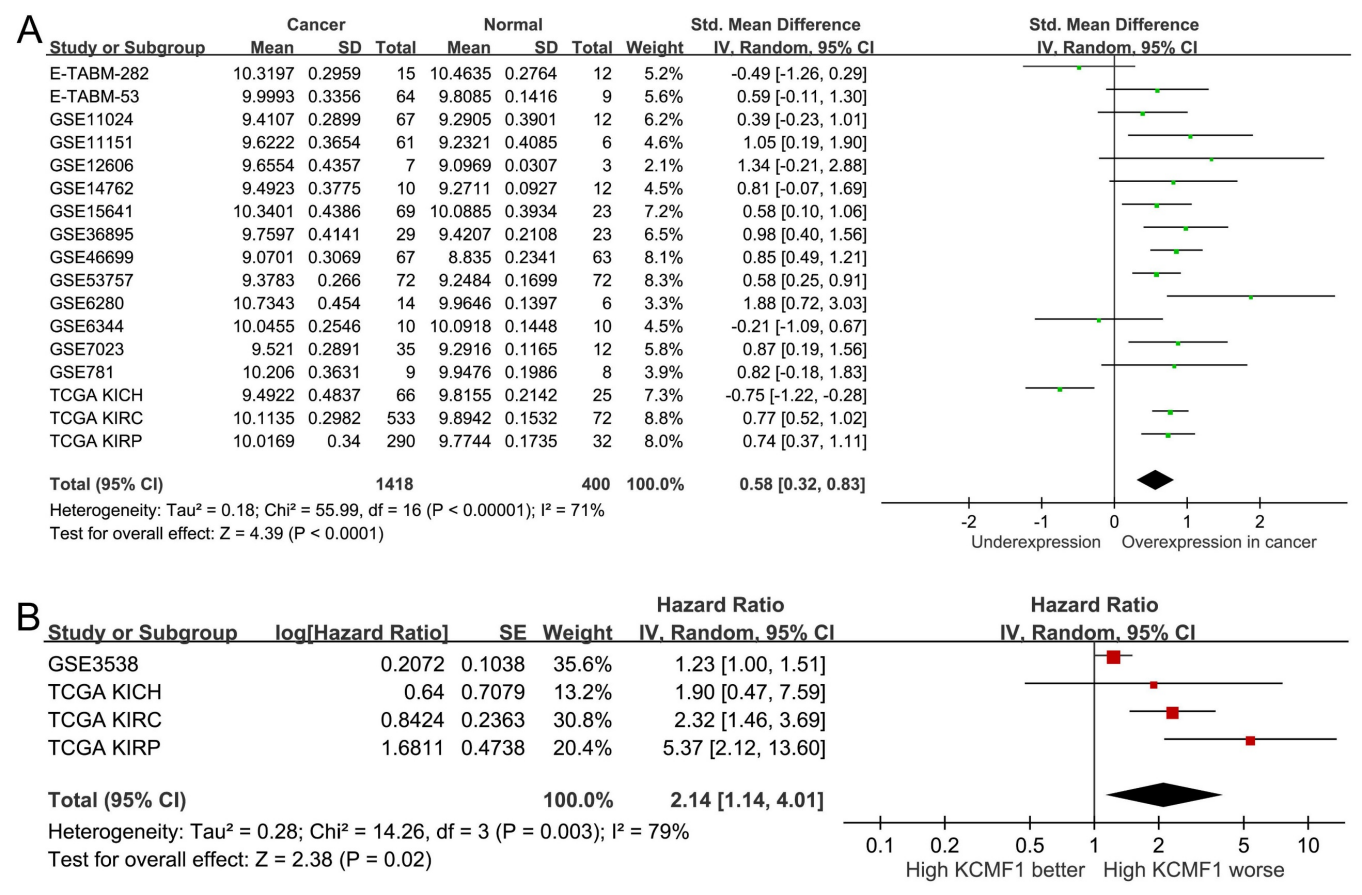
Expression profile and prognostic value of *KCMF1* in kidney cancer. (A) Forest plot summarizing the standardized mean differences in *KCMF1* expression between kidney cancer and normal tissues across 17 independent datasets. (B) Forest plot of the hazard ratio for the association between *KCMF1* expression and patient survival across four datasets. CI, confidence interval; df, degrees of freedom; IV, inverse variance; KICH, kidney chromophobe; KIRC, kidney renal clear cell carcinoma; KIRP, kidney renal papillary cell carcinoma; SD, standard deviation; SE, standard error; Std, standardized; TCGA, The Cancer Genome Atlas.

To validate the clinical relevance of *KCMF1*, publicly-available transcriptomic datasets were analyzed. A pooled analysis of 17 independent studies (comprising 1,418 RCC and 400 adjacent normal tissues) confirmed that *KCMF1* was upregulated in tumor tissues compared to normal controls (SMD = 0.58, 95% CI = 0.32-0.83, *p* < 0.001; Figure 3A). Furthermore, survival analysis across four kidney cancer datasets indicated that elevated *KCMF1* expression was associated with poor prognosis (HR = 2.14, 95% CI = 1.14-4.01, *p* = 0.02; Figure 3B).

**Figure 4:**
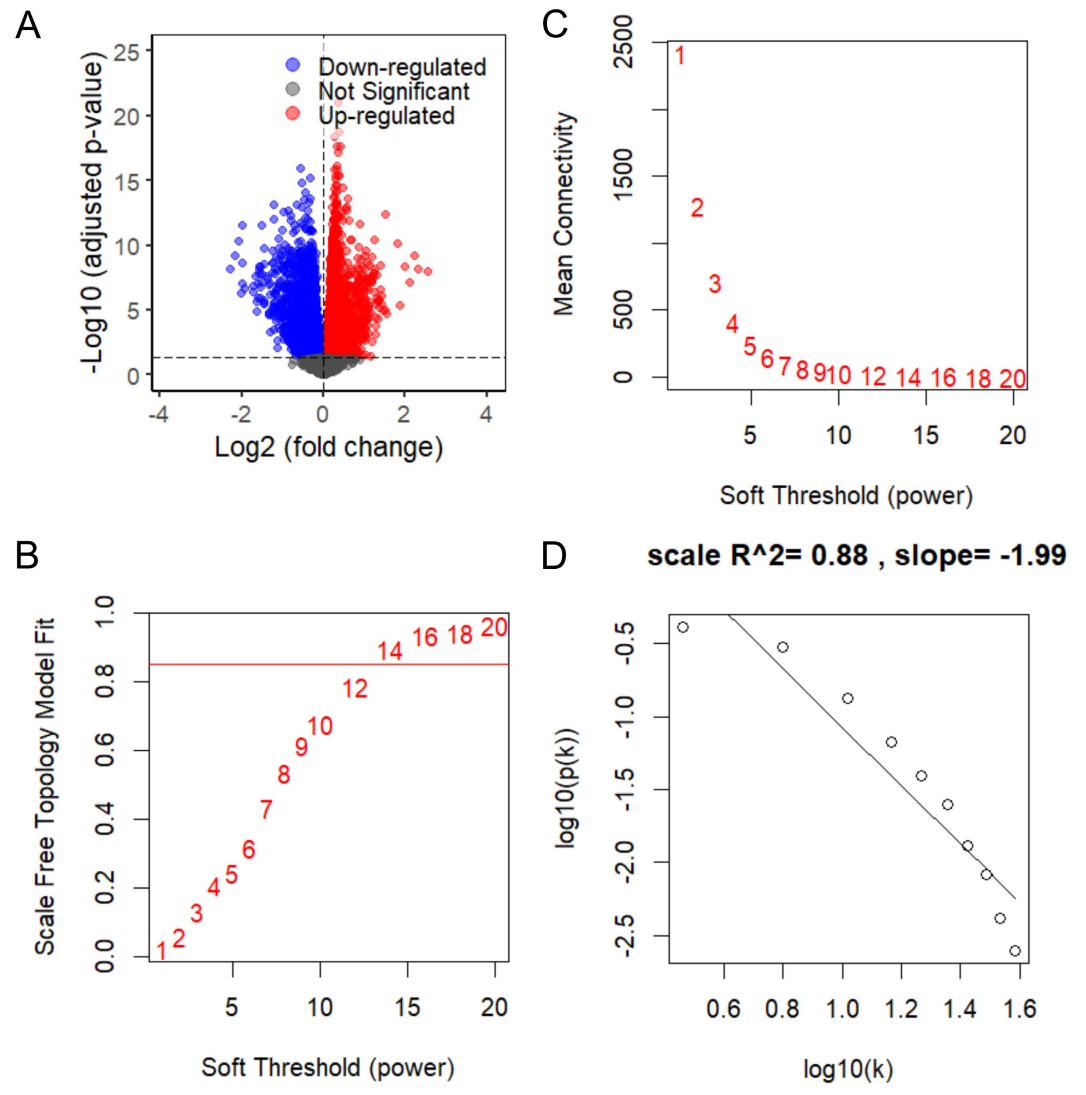
Weighted gene co-expression network analysis of *KCMF1* in kidney cancer. (A) Volcano plot of differentially expressed genes between high- and low-*KCMF1* expression groups in The Cancer Genome Atlas kidney renal clear cell carcinoma cohort. Red and blue dots represent the upregulated and downregulated genes, respectively. (B) Analysis of scale-free fit index for various soft-thresholding powers, β. (C) Analysis of mean connectivity across different soft thresholding powers. (D) Evaluation of scale-free topology fit at the selected soft-thresholding power of β = 14.

To elucidate the biological mechanisms underlying *KCMF1* upregulation in RCC, differential expression analysis followed by WGCNA was conducted using TCGA-KIRC RNA seq data. Stratification of patients by median *KCMF1* expression yielded 7,906 DEGs, comprising 3,910 upregulated and 3,996 downregulated genes (Figure 4A). The DEGs were used to construct a co-expression network. Scale independence and mean connectivity analyses determined that a soft-thresholding power of β = 14 provided the optimal scale-free topology fit (index = 0.88; Figure 4B-D).

**Figure 5:**
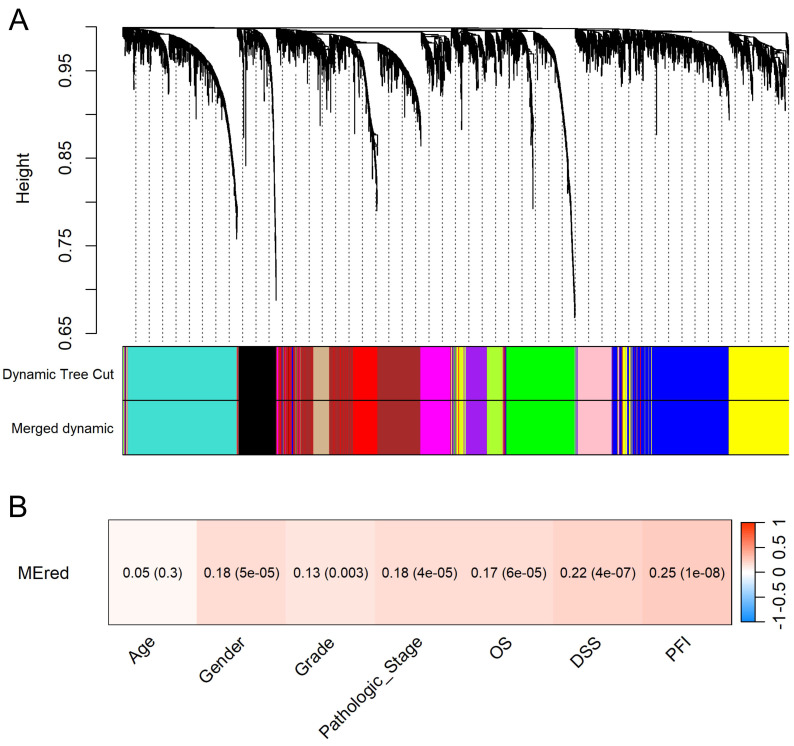
Identification and clinical relevance of the *KCMF1*-associated gene module. (A) Cluster dendrogram of differentially expressed genes based on topological overlap. Genes were grouped into color-coded modules using the dynamic tree cut method. Modules with high similarity were merged using a dissimilarity cut-off height of 0.25. (B) Module-trait relationship heatmap. Each cell displays the correlation coefficient between the *KCMF1*-containing red module eigengene and the clinical traits, with *p*-values in parentheses. The color scale represents the correlation strength and direction (red indicates a positive correlation). OS, overall survival; DSS, disease-specific survival; PFI, progression-free interval.

Hierarchical clustering identified 13 distinct gene modules (Figure 5A). The clinical relevance of the red module containing *KCMF1* was interrogated. Module-trait relationship analysis demonstrated that the red module was positively correlated with tumor grade, pathologic stage, and patient survival outcomes (r ≥ 0.13, *p* ≤ 0.003; Figure 5B), suggesting a role in tumor progression.

**Figure 6:**
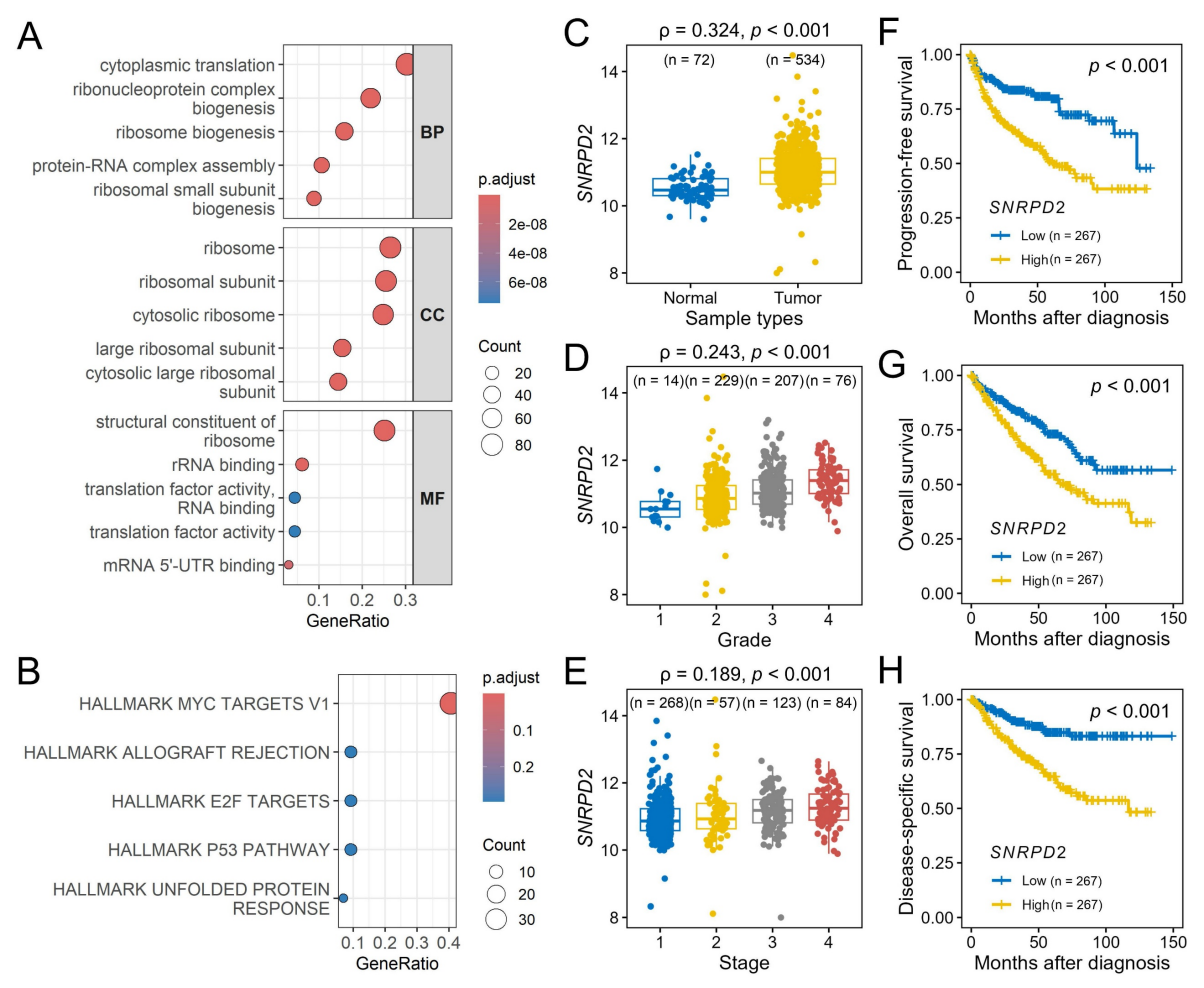
Functional enrichment and hub gene analysis of the red module. (A) Gene Ontology enrichment analysis of the red module categorized by biological process (BP), cellular component (CC), and molecular function (MF). (B) Hallmark pathway enrichment analysis for the red module genes; the top five enriched terms are shown. (C-H) Validation of hub gene, *SNRPD2*, in the Cancer Genome Atlas kidney renal clear-cell carcinoma dataset. *SNRPD2* expression levels in (C) tumor vs. normal tissues, (D) across tumor grades, and (E) across pathological stages. Kaplan-Meier survival curves comparing low and high *SNRPD2* expression for (F) progression-free survival, (G) overall survival, and (H) disease-specific survival. ρ, Spearman's rank correlation coefficient. Numbers in parentheses indicate sample size per subgroup.

Functional enrichment analysis of the red module indicated a predominance of genes involved in the translational machinery. GO analysis indicated enrichment in cytoplasmic translation and ribonucleoprotein complex biogenesis (biological process), ribosome and ribosomal subunits (cellular component), structural constituents of the ribosome and RNA-binding (molecular function) (Figure 6A). Hallmark pathway analysis identified MYC targets v1 as the most significantly enriched pathway (Figure 6B).

Within the MYC targets v1 set, *SNRPD2* emerged as the central hub gene of the red module, exhibiting the highest module membership (0.818) and gene significance for disease-specific survival (0.296). Consistent with their co-membership in the identified pathogenic regulatory network, *SNRPD2* expression exhibited a significant positive correlation with *KCMF1* levels (r = 0.177, *p* < 0.001). Validation using the TCGA-KIRC dataset confirmed that *SNRPD2* was upregulated in RCC tissues (*p* < 0.001; Figure 6C) and positively correlated with advanced tumor grade and stage (*p* < 0.001; Figure 6D-E). Kaplan-Meier analysis indicated that high *SNRPD2* expression was predictive of significantly shorter progression-free, overall, and disease-specific survivals (*p* < 0.001; Figure 6F-H).

## Discussion

To our knowledge, this is the first study to systematically investigate the impact of genetic variants within the ZZ-type zinc finger family on the susceptibility to and progression of RCC. By integrating germline genotyping with transcriptomic network analysis, *KCMF1* was identified as a novel oncogenic driver in RCC. The primary findings highlight the Asian-specific variant, *KCMF1* rs146409312, as a potent susceptibility locus, conferring a greater than three-fold increase in RCC risk, an association that persists independent of established clinical covariates. In addition to genetic susceptibility, *KCMF1* was consistently upregulated in malignant tissues and serves as a predictor of poor clinical outcomes. Using WGCNA, a potential pathogenic mechanism was elucidated wherein *KCMF1* overexpression orchestrated a transcriptional network involved in ribosome biogenesis and MYC signaling, ultimately converging on the critical hub gene, *SNRPD2*. Collectively, these findings bridge the gap between heritable genetic risk and tumor biology, suggesting that *KCMF1* plays a multifaceted role in renal carcinogenesis.

Functional annotation of the 3′ region variant rs146409312 indicated a chromatin landscape characteristic of active promoters and enhancers, marked by DNase I hypersensitivity, RNA polymerase II occupancy, and enriched transcription factor-binding motifs. These features strongly suggest that rs146409312 modulates *KCMF1* transcriptional activity. Matched germline genotype-expression datasets from normal kidney tissue are currently unavailable at sufficient scale. Therefore, lymphoblastoid cell lines derived from HapMap individuals were used as a validated model for germline eQTL analysis, enabling assessment of constitutive regulatory effects of rs146409312 on *KCMF1* expression without confounding somatic alterations present in tumor tissues. Although the eQTL analysis in the HapMap East Asian cohort yielded only a trend regarding the effect of risk alleles on mRNA levels, which is likely attributable to the limited sample size of minor allele carriers (n = 9, MAF = 0.04), the oncogenic potential of *KCMF1* is supported by physiological evidence. Analysis of the TCGA-KIRC cohort revealed that *KCMF1* harbors an exceptionally low somatic mutation frequency (approximately 0.2%) in RCC, in sharp contrast to canonical driver genes such as *VHL* (49.9%). This mutational profile indicates that *KCMF1* is unlikely to function as a classical driver through recurrent coding alterations. Instead, these data support a model in which *KCMF1* contributes to RCC tumorigenesis predominantly through germline regulatory variation and transcriptional dysregulation. *KCMF1* functions as a novel E3 ubiquitin ligase that forms a complex with ubiquitin-conjugating enzyme E2 and ubiquitin protein ligase E3 component, N-recognin 4, to orchestrate lysosome-mediated degradation and autophagy, particularly under hypoxic stress 26. *KCMF1* is overexpressed during pancreatic cancer development, where it enhances cell proliferation, migration, and invasion by upregulating cell cycle regulators including cyclin D and cyclin dependent kinase 4, and *KCMF1* knockdown in transgenic mice substantially reduced premalignant lesions and prevented pancreatic cancer formation 27. In RCC, *KCMF1* regulates autophagy and ion channel function, with disrupted *KCMF1*-associated ubiquitin ligase formation and altered ionic concentrations observed in tumor tissues 28. *KCMF1* overexpression promotes cancer progression through the KCMF1/FUS/centromere protein T axis, which activates JNK signaling pathways and enhances RCC proliferation, invasion, and metastasis 11. Thus, *KCMF1* overexpression rewires the ubiquitin-proteasome and stress response pathways to promote tumor growth. *KCMF1* has been implicated in autophagy and lysosome-mediated degradation, cellular processes that are mechanistically linked to ferroptosis. Consistent with this, ferroptosis pathway activity showed a modest correlation with *KCMF1* expression and tumor grade in RCC (data not shown). However, ferroptosis-related gene sets were not enriched within the *KCMF1*-associated co-expression module, nor were ferroptosis scores associated with disease stage or patient survival, suggesting that ferroptosis is unlikely to represent the primary downstream effector of *KCMF1* in RCC. Instead, our data indicate that *KCMF1*-driven tumor progression is more strongly mediated through MYC-dependent transcriptional and translational reprogramming.

To elucidate the downstream effectors of this oncogenic signaling, WGCNA identified a robust enrichment of the MYC target v1 gene set within the *KCMF1*-associated network, with *SNRPD2* emerging as a critical hub gene. This observation is mechanistically compelling, as *SNRPD2* is a direct transcriptional target of MYC: chromatin immunoprecipitation and promoter analyses across multiple cancer models have demonstrated MYC binding to E-box elements within the *SNRPD2* promoter, resulting in strong transcriptional activation in MYC-driven tumors 29. Functionally, MYC-mediated upregulation of *SNRPD2* supports the increased spliceosomal and translational demands imposed by MYC-driven proliferation, positioning *SNRPD2* as a critical executor of MYC oncogenic programs 30. Importantly, *SNRPD2* does not function solely as a passive MYC effector; rather, it has been shown to reinforce MYC signaling through post-transcriptional mechanisms, including the regulation of oncogenic RNA splicing and the promotion of *MYC* mRNA nuclear export via spliceosome-dependent control of RNA helicase isoforms 29. This establishes a feed-forward MYC-SNRPD2 regulatory loop, whereby MYC transcriptionally induces SNRPD2, and elevated SNRPD2 activity sustains MYC protein abundance and transcriptional output.

*SNRPD2* encodes the core spliceosome protein, which is a structural component of the U2 spliceosome that is essential for pre-mRNA splicing and mature mRNA production. Pan-cancer analysis indicated *SNRPD2* overexpression in almost all solid tumor types, with high expression associated with poor prognosis in multiple cancers, including colorectal, lung, hepatocellular carcinoma (HCC), and RCC 31. In colorectal cancer, *SNRPD2* is upregulated and disrupts liquid-liquid phase separation of poly(A) binding protein nuclear 1 by interacting with its glutamic-proline domain, leading to shortened 3′ untranslated region of catenin beta interacting protein 1 through alternative polyadenylation, thereby promoting cell proliferation and migration 32. Similarly, in lung adenocarcinoma, elevated *SNRPD2* expression is correlated with worse overall survival, particularly in nonsmoking patients, in whom high *SNRPD2* expression confers a substantially increased mortality risk 33. Cancer cells exhibit selective vulnerability to *SNRPD2* depletion, with silencing experiments showing cancer-specific lethality, whereas normal cells tolerate reduced *SNRPD2* expression, establishing *SNRPD2* as a promising cancer-selective therapeutic target 31. In HCC, *SNRPD2* dictates the splicing of critical DNA repair genes, such as *BRCA1* and FA complementation group D2, creating a vulnerability where *SNRPD2* depletion induces exon skipping and sensitizes cells to poly(ADP-ribose) polymerase inhibitors 30. Our integrative analyses suggest that *KCMF1* overexpression, potentially driven by the risk allele, establishes a cellular context characterized by elevated MYC activity. Within this MYC-active transcriptional network, *SNRPD2* is consistently upregulated. Increased *SNRPD2* expression facilitates efficient RNA splicing and ribosome biogenesis, thereby supporting enhanced protein synthesis and cellular proliferation. Clinically, high *SNRPD2* expression correlates with advanced tumor grade, higher pathological stage, and poor survival outcomes. Collectively, these findings underscore the biological complexity of the KCMF1-SNRPD2 axis and support further investigation of its mechanistic contributions to RCC progression and its potential clinical utility as both a prognostic biomarker and a therapeutic target.

The primary strength of this study is its integrative design, which bridges germline genetic susceptibility with tumor-specific transcriptomic networks. In addition to traditional single-locus association studies, WGCNA was used to map the downstream KCMF1-SNRPD2 axis, providing a plausible molecular mechanism for the observed risk. Adjustment for comprehensive clinical covariates, including comorbidities and lifestyle factors, enhanced the reliability of this association. However, this study has several limitations that must be acknowledged. The sample size of 630 participants, which is sufficient for candidate gene analysis, was relatively modest compared to large-scale genome-wide studies, potentially limiting the detection of rare variants. Because rs146409312 is an Asian-specific variant, the findings may not be generalizable to non-Asian populations, highlighting the need for validation in diverse ethnic cohorts. Although the bioinformatic analyses strongly suggest a regulatory link between *KCMF1* and *SNRPD2*, *in vitro* or *in vivo* functional experiments are required to definitively establish biological causality.

## Conclusion

A novel *KCMF1*-driven oncogenic axis was identified in RCC, linking the germline variant, rs146409312, to tumor aggressiveness via an *SNRPD2*-mediated network. The identification of rs146409312 as an Asian-specific risk allele underscores the importance of population-specific genetic screening in the era of precision medicine. Clinically, these findings suggest that stratifying patients based on *KCMF1* genotype/expression status could refine diagnostic/prognostic models and identify high-risk individuals who may benefit from intensified surveillance. Elucidation of the downstream SNRPD2 and MYC target signaling pathways provides a rationale for exploring therapeutic strategies targeting this specific molecular network. Future functional studies are warranted to fully dissect the KCMF1-SNRPD2 regulatory hierarchy and validate these biomarkers in prospective, multi-ethnic cohorts.

## Supplementary Material

Supplementary table.

## Figures and Tables

**Table 1:** Association of *KCMF1* rs146409312 with the risk of renal cell carcinoma

Genotype	Controls, n (%)	Patients, n (%)	OR (95% CI)	*p*	*q*	OR (95% CI)^a^	*p* ^a^
TT	304 (96.2)	275 (88.1)	1.00			1.00	
TA	12 (3.8)	36 (11.5)	3.32 (1.69-6.50)	0.0005		3.16 (1.53-6.52)	0.002
AA	0 (0.0)	1 (0.3)	-	-		-	-
TA/AA	12 (3.8)	37 (11.9)	3.41 (1.74-6.67)	0.0003		3.23 (1.57-6.65)	0.001
Trend			3.38 (1.74-6.58)	0.0003	0.048	3.22 (1.57-6.58)	0.001

Abbreviations: OR, odds ratio; CI, confidence interval. ^a^ORs were adjusted for sex, age, body mass index, smoking status, alcohol intake, and histories of diabetes and hypertension.
