# Supplementary material for: Integrated analysis uncovers KCMF1 genetic susceptibility and the SNRPD2 axis in renal cell carcinoma
Source: Int J Med Sci. 2026 Feb 18;23(4):1198–208. doi: 10.7150/ijms.129880 (PMC13048855; doi:10.7150/ijms.129880)
Supplement: Supplementary file 1 — Supplementary table. [file ijmsv23p1198s1.pdf]

Table S1. Clinical and demographic characteristics of healthy controls and patients with renal cell carcinoma

Characteristic	Controls (n = 318)	Patients (n = 312)	*p*
Sex, n (%)			
Male	210 (66.0)	209 (67.0)	0.801
Female	108 (34.0)	103 (33.0)	
Age, y			
Median (IQR)	57 (47-68)	57 (48-68)	
≤57	162 (50.9)	160 (51.3)	0.932
>57	156 (49.1)	152 (48.7)	
Body mass index, kg/m^2			
Median (IQR)	24.6 (22.6-26.7)	24.4 (22.3-27.6)	
≤25	163 (57.0)	172 (55.5)	0.711
>25	123 (43.0)	138 (44.5)	
Smoking status, n (%)			
Ever	105 (33.0)	114 (36.5)	0.354
Never	213 (67.0)	198 (63.5)	
Alcohol intake, n (%)			
Ever	135 (42.5)	76 (24.4)	<0.001
Never	183 (57.5)	236 (75.6)	
Hypertension, n (%)			
Yes	78 (24.9)	134 (43.1)	<0.001
No	235 (75.1)	177 (56.9)	
Diabetes, n (%)			
Yes	20 (6.3)	62 (19.9)	<0.001
No	296 (93.7)	249 (80.1)	
Stage, n (%)			
I-II		240 (81.4)	
III-IV		55 (18.6)	
Grade, n (%)			
I-II		206 (75.2)	
III-IV		68 (24.8)	

Abbreviations: IQR, interquartile range.

Subtotals do not sum to n of patients due to missing data.
